# Managing Obesity in Lockdown: Survey of Health Behaviors and Telemedicine

**DOI:** 10.3390/nu13041359

**Published:** 2021-04-19

**Authors:** Noga C. Minsky, Dafna Pachter, Galia Zacay, Naama Chishlevitz, Miriam Ben-Hamo, Dana Weiner, Gabriella Segal-Lieberman

**Affiliations:** 1 Chaim Sheba Medical Center, Division of Endocrinology, Diabetes and Metabolism, Tel-HaShomer, Ramat-Gan 5266202, Israel; dafna.pachter@sheba.gov.il (D.P.); Naama.Chishlevitz@sheba.gov.il (N.C.); gabriella.lieberman@sheba.gov.il (G.S.-L.); 2 Sackler Faculty of Medicine, University of Tel Aviv, Tel Aviv 6997801, Israel; galia_zacay@meuhedet.co.il (G.Z.); benhamo@mail.tau.ac.il (M.B.-H.); 3 Chaim Sheba Medical Center, Department of Nutrition, Tel-Hashomer, Ramat-Gan 5266202, Israel; dana.weiner@sheba.gov.il; 4 Department of Family Medicine, Meuhedet Health Maintenance Organization, Tel Aviv 6203854, Israel

**Keywords:** COVID-19, SARS-CoV-2, dietary habits, exercise, mood, quarantine, social distancing, telemedicine, obesity, weight gain

## Abstract

Since the outbreak of COVID-19, billions of people have gone into lockdown, facing pandemic related challenges that engender weight gain, especially in the obese. We report the results of an online survey, conducted during Israel’s first quarantine, of 279 adults treated in hospital-based obesity clinics with counseling, medications, surgery, endoscopic procedures, or any combination of these for weight loss. In this study, we assessed the association between changes in dietary and lifestyle habits and body weight, and the benefits of receiving weight management care remotely through telemedicine during lockdown. Compared to patients not receiving obesity care via telemedicine, patients receiving this care were more likely to lose weight (OR, 2.79; *p* = 0.042) and also to increase participation in exercise (OR, 2.4; *p* = 0.022). While 40% of respondents reported consuming more sweet or salty processed snacks and 33% reported less vegetables and fruits, 65% reported more homemade foods. At the same time, 40% of respondents reported a reduction in exercise and 52% reported a decline in mood. Alterations in these eating patterns, as well as in exercise habits and mood, were significantly associated with weight changes. This study highlights that lockdown affects health behaviors associated with weight change, and advocates for the use of telemedicine to provide ongoing obesity care during future quarantines in order to promote weight loss and prevent weight gain.

## 1. Introduction

The SARS-CoV-2 pandemic has led to an unprecedented global response, in which countries around the world, including Israel, declared lockdowns and imposed restrictions to limit the spread of disease. These measures have led to significant changes in human behavior, affecting dietary habits, physical activity, occupational status, and mental health, all of which can affect body weight.

A number of studies have demonstrated an association between these restrictions and weight gain, which may be attributed to several factors [1,2,3,4]. It is known that people tend to eat excessively when they are unable to express their feelings, which is a characteristic of lockdown [5]. Moreover, restrictions on mobility and the closure of fitness facilities led to a decrease in daily physical activity [6]. In addition, prolonged confinement and the combination of working and studying from home are associated with an increase in screen time [7], and prolonged screen time can have a negative impact on eating behaviors and weight [8]. The current pandemic has been accompanied by uncertainty, stress and social distancing, and accordingly, a decline in mood has been reported [9,10]. A large prospective study has shown that depression and emotional eating are associated with weight gain [11]. In general, people with obesity (PwO) display more problematic eating behaviors, such as eating in the absence of hunger [12], which may be exacerbated during confinement to the home with unlimited access to food.

A fear to leave the home amid the pandemic and reduced access to routine care have both led to a reduction in medical visits for chronic conditions, including obesity. This has compounded the risk of weight gain, and is especially problematic given the fact that obesity is an independent risk factor for COVID-19 related critical illness and mortality [13]. This presented a challenge for the medical community, which sought novel solutions to provide care remotely during lockdown. In turn, this led to a surge in the use of telemedicine to facilitate access to care for chronic conditions, predominantly in the form of virtual clinics between patients and providers [14]. It has previously been demonstrated that video visits with physicians and dietitians can be effective in driving weight loss compared to standard care [15].

In this cross-sectional survey study, we explored the relationships between diet and other health behaviors, as well as telemedicine use during the lockdown in Israel in adult PwO followed in hospital-based obesity clinics. Behavior changes and population subgroup characteristics, including demographics, obesity treatments and virtual care, were examined for associations with reported weight changes. Understanding the interplay between dietary and lifestyle habits, weight lost treatments, and telemedicine in treating PwO during lockdown provides insights about the role that healthcare professionals can play in treating obesity during quarantines in the future.

## 2. Materials and Methods

### 2.1. Participants

At Sheba Medical Center, an invitation to complete the survey was sent to the 2602 adults who, over the past two years, visited the multidisciplinary weight management clinic or the affiliated obesity clinic. Respondents were excluded from the study if they endorsed pregnancy in their survey responses.

### 2.2. Clinical Services

The multidisciplinary weight management clinic is headed by endocrinology and is staffed by dietitians, endocrinologists, psychologists, and a sports medicine doctor and nurse. Patients followed in this clinic are offered a wide range of weight loss therapies including counseling, medications, referral to bariatric surgery, and referral for endoscopic sleeve gastroplasty. The obesity clinic is headed by surgery and is staffed by bariatric surgeons, dietitians, nurses, and psychologists. In both clinics, telemedicine for obesity management is offered in the form of video calls via the Datos Health Ltd. platform. Video calls typically last 20–40 min.

### 2.3. Procedures

We sent a link to the online Hebrew language structured questionnaire (Supplementary Materials) via telephone text messaging. The study was conducted according to the guidelines of the Declaration of Helsinki, and was approved by the Institutional Review Board of Sheba Medical Center (7218-20-SMC). Informed consent was obtained from all subjects involved in the study. Surveyed patients were asked to report on changes experienced since lockdown, which began on 11 March 2020. The survey was sent on 7 May and responses were collected through 18 May 2020. Meanwhile, restrictions were very gradually and partially eased over the month of May. Question topics included demographics, anthropometric information, eating habits, physical activity, sleep, mental health, obesity treatments, and medical visits. Patients were asked to compare their current state during lockdown to their state before lockdown. Subjects had the option to complete the survey without answering all questions. 

### 2.4. Statistical Analysis

Since there were a limited number of responses, we had to restrict the number of independent variables in our multivariate analysis, and therefore, we utilized a composite dietary habit score instead of analyzing each dietary habit separately. This score was calculated based on responses to questions regarding changes in consumption of sweet or salty processed snacks, fruits and vegetables, and homemade foods. Each response was graded on a scale of 1–3, with “1” indicating a deterioration in the dietary habit, “2” indicating no change, and “3” indicating an improved dietary habit. These values were summed to a composite score of 3–9. A composite score of 3–5 was considered as “deterioration”, 6 as “no change” and 7–9 as “improvement”. Univariate chi-square analysis, univariate ANOVA and multivariate logistic regression were conducted and a two-tailed *p*-value < 0.05 was considered significant.

## 3. Results

### 3.1. Baseline Characteristics

There were 279 valid responses, with two patients excluded because of pregnancy. The average age of respondents was 53 ± 13 years and 69% were female. The mean reported weight before lockdown was 91.6 ± 23.8 kg and the mean estimated BMI was 33.9 ± 7.6 kg/m^2^ (IQR 28.4–39.0). One hundred and eighty-one patients (65%) were from Israel’s Central District, while 98 were from other parts of the country (21% Judaean Foothills, 11% Negev, and 2% northern districts). Overall, 49% of patients reported that their primary weight loss intervention was invasive (43% bariatric surgery, 6% endoscopic sleeve gastroplasty), 15% reported a pharmacologic intervention, and 36% reported neither type of intervention, presumably having received counseling alone (data not reported in tables).

### 3.2. Weight Change

Two hundred and forty-three patients reported their weight at the time of receiving the questionnaire, with a mean change in weight of −0.1 kg ± 4.8 kg. For the purpose of analysis, given that lockdown lasted under two months, respondents were divided into tertiles based on their reported weight change: “weight loss” > −0.4 kg, “no weight change” −0.4 kg to +1.5 kg, and “weight gain” > +1.5 kg. 

There were significant associations between changes in weight and reported changes in mood, weekly exercise time, and composite dietary habit score that occurred during lockdown according to the univariate analysis (Table 1). Specifically, 55% of respondents who exercised more during lockdown were in the weight loss group, while only 30% of respondents who exercised the same or less in the weight loss group. Additionally, of those who reported a decline in mood, 43% were in the weight gain group, but only 23% of patients who reported no change or an improvement in mood were allocated to this group. Lastly, 49% of those who had improvement in their dietary habit score were in the weight loss group, compared with only 29% whose score was unchanged, and 22% whose composite score deteriorated. Furthermore, significant associations were observed between reported weight change and primary type of weight loss treatment received.

### 3.3. Telemedicine Utilization

Fifty-one patients (18%) received virtual care, including 31 patients who indicated visits with a dietitian, 33 with a physician, and 9 with a mental health professional. Only 14 respondents had video visits with our providers before lockdown. 

There were a number of significant differences between the 51 respondents who utilized our telemedicine services over lockdown and the 228 respondents who did not (Table 2). Compared to patients who received counseling alone, patients who indicated invasive procedures (bariatric surgery or endoscopic sleeve gastroplasty) were less likely to receive virtual care during this period (OR, 0.39; 95% CI 0.18–0.84). On the other hand, patients who indicated medications as their primary treatment for weight loss were more likely to take advantage of virtual care (OR, 3.5; 95% CI 1.60–7.67). 

Patients who were followed via video visits during lockdown had a significantly higher mean baseline weight (98 versus 90 kg; *p* = 0.02). Over the approximately two month period, telemedicine users achieved a mean weight loss of 1.3 ± 5.2 kg, compared to a mean weight gain of 0.18 ± 4.6 kg in nonusers (*p* = 0.07) (Table 2). There were no statistically significant differences between age, gender, reported mood changes, or composite dietary habit scores between patients who did or did not have video visits at our center during confinement. Respondents who received virtual obesity care were more than twice as likely to report increased weekly exercise time during lockdown (OR, 2.4; 95% CI 1.12–5.00; *p* = 0.022) (data not reported in tables). 

According to logistic regression analysis controlling for sex, age, treatment and virtual services use, patients who had weight management video visits during lockdown had significantly higher odds for being in the weight loss group compared to the weight gain group, with an odds ratio of 2.79 (95% CI 1.04–7.46; *p* = 0.042) (Table 3). According to this analysis, gender was not associated with a difference in odds for weight loss, but for each additional 10 years of age, there was a trend for weight loss (OR 1.29; 95% CI 0.97–1.7; *p* = 0.082).

Thirty-five percent of respondents indicated that they would be interested in continuing to conduct tele-nutrition visits after cessation of COVID-19-related restrictions, including 42 (58%) of patients from Central Israel, and 30 (15%) from other districts.

### 3.4. Lifestyle and Well-Being

**Figure 1:**
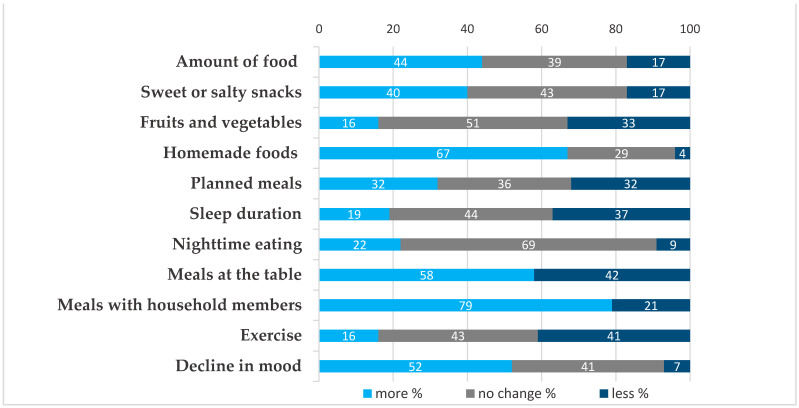
Responses to selected questions regarding changes during lockdown.

During lockdown, a number of changes in lifestyle were reported (Figure 1). In terms of dietary habits, 44% of patients reported consuming more food overall, 40% more sweet or salty processed snacks, 22% more nighttime meals, and 33% less vegetables and fruits. In contrast, there were some positive changes reported in dietary patterns related to spending more time at home, with 65% consuming more homemade foods and 79% reporting eating more often with members of their household. While 40% of respondents reported a reduction in weekly exercise time, 16% reported increased exercise. Fifty-two percent reported a decline in mood and less night-time sleep was recorded by 37%. Sixty patients (22%) of patients became unemployed (data not in figure).

### 3.5. Weight Loss Treatments

Respondents were asked to select their most pertinent therapeutic modality for weight loss. Forty-five percent selected bariatric surgery (19% had surgery within the past year, and 81% had surgery previously), 15% received weight loss medications, 6% underwent endoscopic sleeve gastroplasty, and 34% selected none of the above. 

Weight changes were compared between patients receiving and not receiving pharmacologic or invasive obesity interventions, with the latter group presumably receiving counseling alone. According to logistic regression analysis, which compared the defined group of patients who lost weight to the group of patients who gained weight, controlling for sex, age, treatment and use of virtual services, therapies other than bariatric surgery did not demonstrate a statistically significant association with weight change. Patients who underwent bariatric surgery within the past 12 months prior to the confinement period were significantly more likely to experience weight loss than patients receiving counseling alone (OR, 22.37; 95% CI 2.58–193.87). On the other hand, patients who underwent bariatric surgery more than 12 months previously were less likely to experience weight loss than patients receiving counseling alone who had never undergone bariatric surgery (OR, 0.433; 95% CI 0.92–0.98) (Table 3). 

## 4. Discussion

This is the first study to demonstrate an association between weight loss and the use of telemedicine for obesity management during lockdown. According to our survey of 279 PwO in Israel, receiving multidisciplinary obesity care via telemedicine conferred a nearly three-fold increased odds (OR 2.79) for being in a weight loss versus a weight gain category, according to a logistic regression analysis controlling for sex, age, and obesity treatment type. This difference is remarkable given that the first lockdown in Israel lasted only around two months. Given that overweight and obese subjects tend to gain weight more frequently during lockdown, the potential impact of telemedicine in weight management is far-reaching [16]. 

There was a more than 3.5-fold rise in the use of telemedicine in our center during the first quarantine, reflecting the rapid endorsement of this mode of healthcare delivery by both patients and providers. It should be noted that in parallel, there was a decline in in-person visits given the limited transportation and travel restrictions for what was deemed “non-essential” medical care. In anticipation of this situation, the hospital rapidly expanded telemedicine services, which were available only for dietitian visits in our clinic before March of 2020. Virtual care became synonymous with receiving ongoing care. Even though most respondents resided in the same district as our medical center, thirty-five percent of patients indicated that they would be interested in continuing to conduct tele-nutrition visits after cessation of pandemic related restrictions. Therefore, continuing to offer expanded telemedicine services post lockdown is expected to facilitate and enhance care for patients near and far.

Several factors may explain why patients actively receiving obesity care during lockdown were more likely to experience weight loss in our study. Patients followed with video visits were more than twice as likely to report increased exercise, which could reflect the positive effects of actively receiving care through telemedicine. While telemedicine use was not associated with a better composite dietary habit score, it is possible that our study was underpowered to detect an improvement in different individual dietary habits that ultimately led to more weight loss. Patients who had video visits during lockdown had a higher reported baseline BMI, and therefore, may have been more motivated to lose weight. Those receiving weight loss medications were more likely to utilize telemedicine, and those receiving invasive weight loss procedures (bariatric surgery or endoscopic sleeve gastroplasty) were less likely to utilize telemedicine. Therefore, the higher baseline BMI for patients receiving virtual care could be related to the fact that they received a less aggressive weight loss intervention. Lastly, patients actively receiving follow up care during lockdown could potentially be more likely to be in the weight loss phase of obesity care, rather than the weight maintenance phase, and this could have contributed to the fact that patients receiving virtual care were more likely to lose weight. 

Telemedicine has been demonstrated, according to two comprehensive reviews of pediatric studies, to be non-inferior to in-person treatment programs, as measured by weight loss, with high rates of patients satisfaction [17,18]. While less has been published about the use of telemedicine to treat obesity in adults, it has been suggested as a novel and powerful way to deliver comprehensive patient centered care, and as a tool to increase access to bariatric surgery, where less than 1% of eligible patients undergo this procedure, and half of those who initiate a treatment program drop out [19]. 

Besides the use of telemedicine, there were a number of factors that were associated with healthier weight change trends over lockdown according to univariate analysis in our study. Those who increased weekly exercise time were more likely to lose weight overall, and this was similarly reported surveys from China [20] and Spain [2]. Respondents who had a positive change in their dietary habit score (based on consumption of sweet and salty processed snacks, fruits, vegetables and homemade food) reported weight loss, while those that had a negative change gained weight and this difference was significant. A deterioration in dietary habits was associated with weight gain in surveys form around the word during quarantine, including snacking [2], night eating [21], “bingeing on food” [22], and consumption of sugary drinks, pastries and fried foods [3]. Furthermore, in our study an improved mood was associated with weight loss, and a decline in mood was associated with weight gain. Though causation cannot be established, this finding is consistent with a survey-based study conducted in the University of Southern California, where college students who had higher depression scale scores were more likely to use food as a coping behavior during the pandemic, and among students with a heavier baseline weight, this type of eating was associated with weight gain [23]. 

The primary obesity therapy treatment indicated by our respondents was also associated with differential weight changes during lockdown. As expected, patients who underwent bariatric surgery within the past year were more likely to lose weight during confinement compared to those who received counseling alone. On the other hand, patients who underwent bariatric surgery over a year before lockdown were less likely to lose weight during confinement compared to those who received counseling alone with no history of bariatric surgery. It has been demonstrated that weight regain can be common two years after bariatric surgery [24].

Even though our report sheds light on how pandemic related restrictions affect health habits and weight, and what can be done about it, there are limitations that must be considered. Our cross-sectional study is based on survey results, and therefore, provided data were not verifiable and should be treated as estimates. Self-reported answers may be affected by various biases. For example, as regards the association of weight gain with a decline in mood, patients may be more likely to report a decline in mood after indicating weight gain. Importantly, the patients surveyed were heterogeneous in terms of the types of obesity therapies they received. It must be considered that in certain situations, such as with initiation of weight loss medications or recent bariatric surgery, these therapies can be critical elements in driving acute weight loss and that this may confound the effects of lockdown or the use of telemedicine on weight. In addition, since surveyed PwO were followed in our hospital clinics, selection bias may affect the observed associations. For example, patients who experience weight regain after bariatric surgery are presumably more likely to present to obesity clinics, and therefore, our patients may not accurately represent the entire population of patients who underwent bariatric surgery. Collectively these limitations suggest the need for further studies, which are feasible, given that at the time of this writing, restrictions remain in place in many countries worldwide.

## 5. Conclusions

According to survey responses from PwO during quarantine, reported weight loss was associated with improved dietary habits, increased exercise, and the use of telemedicine to support weight management. Ongoing multidisciplinary care, made possible in the form of video visits, encourages positive lifestyle changes and weight loss despite a trend of deterioration in a number of health habits for many PwO during lockdown. Given convenience, patient preferences, and efficacy, we expect that continued utilization of telemedicine in obesity management will improve outcomes going forward. After this pandemic, there is expected to be a heightened need to address the consequences of uncontrolled weight gain, which has long term global health implications. Telemedicine has quickly been recognized as an invaluable tool during the pandemic, and in agreement with regional physicians [14], we believe its use should be maximized and integrated with face to face visits as we progress beyond the pandemic.

## Figures and Tables

**Table 1:** Characteristics of 243 patients who provided data as regards weight change divided among tertiles of weight change categories.

Characteristics	Weight Loss > −0.4 kg	No Weight Change 0.4 to +1.5 kg	Weight Gain > +1.5 kg	*p*-Value
Overall (n (%))	81 (33)	81 (33)	81 (33)	
Gender				0.67
Women (n (%))	53 (32)	58 (35)	57 (34)	
Men (n (%))	28 (37)	23 (31)	24 (32)	
Mean age (years (SD))	53 (±14)	55 (±12)	50 (±12)	0.11
Mean baseline weight (kg (SD))	94 (±24)	89 (±24)	91 (±23)	0.38
Treatment				0.001
Bariatric over one year ago (n (%))	15 (18)	29 (35)	39 (47)	
Bariatric within past year (n (%))	14 (64)	7 (32)	1 (5)	
Endoscopic sleeve gastroplasty (n (%))	7 (47)	3 (20)	5 (33)	
Pharmacological (n (%))	14 (39)	14 (39)	8 (22)	
Other (n (%))	31 (36)	28 (32)	28 (32)	
Exercise				0.009
More (n (%))	21 (55)	10 (23)	7 (18)	
Same or less (n (%))	56 (30)	64 (35)	67 (36)	
Mood				0.006
No change or improved (n (%))	42 (39)	42 (39)	25 (23)	
Worse (n (%))	34 (30)	31 (27)	49 (43)	
Composite dietary habit score *				<0.001
Improvement (n (%))	40 (49)	26 (32)	16 (20)	
No change (n (%))	19 (29)	28 (43)	18 (28)	
Deterioration (n (%))	21 (22)	27 (28)	47 (50)	

* Consumption of sweet or salty processed snacks, fruits and vegetables and homemade foods.

**Table 2:** Characteristics associated with telemedicine use compared to nonuse in 279 patients.

Characteristic	Using	Not Using	*p*-Value
Overall n (%)	51 (18)	228 (82)	
Gender			0.81
Female (n (%))	36 (19)	157 (81)	
Male (n (%))	15 (17)	71 (83)	
Mean age (years (SD))	52 (14)	53 (12)	0.98
Mean weight baseline (kg (SD))	98 (±24)	90 (±24)	0.02
Mean weight change (Kg (SD))	−1.3 (±5.2)	0.18 (±4.6)	0.07
Treatment			<0.001
Bariatric over one year ago (n (%))	7 (7)	91 (93)	
Bariatric within past year (n (%))	1 (4)	22 (96)	
Endoscopic sleeve gastroplasty (n (%))	4 (25)	12 (75)	
Pharmacological (n (%))	19 (43)	22 (54)	
None of the above (n (%))	20 (20)	81 (80)	
Exercise			0.02
More (n (%))	13 (31)	29 (62)	
Same or less (n (%))	34 (16)	179 (84)	
Mood			0.07
No change or improved (n (%))	26 (22)	95 (79)	
Worse (n (%))	17 (13)	116 (87)	
Composite dietary habit score *			0.467
Improvement (n (%))	20 (21)	76 (79)	
No change (n (%))	14 (21)	54 (79)	
Deterioration (n (%))	17 (15)	97 (85)	

* Consumption of sweet or salty processed snacks, fruits and vegetables and homemade foods.

**Table 3:** Multivariate logistic regression analysis characteristics associated with being in a weight loss versus weight gain category (adjusted for all variables in table).

	Odds Ratio	*p*-Value	95% CI
Telemedicine use	2.79	0.042	1.04–7.48
Female versus male	0.74	0.416	0.35–1.54
Age (each ten-year increase)	1.29	0.082	0.97–1.72
Treatment *			
Bariatric over 12 months ago	0.43	0.044	0.19–0.98
Bariatric with 12 months	22.37	0.005	2.58–193.87
Endoscopic sleeve gastroplasty	1.50	0.543	0.41–5.51
Pharmacological	1.17	0.776	0.40–3.38

* Treatment arms are compared to patients who selected “none of the above [treatments]”, and presumably received counseling alone.

## Data Availability

The data presented in this study are available on request from the corresponding author. The data are not publicly available due to privacy reasons.

## Supplementary Materials

The following are available online at https://www.mdpi.com/article/10.3390/nu13041359/s1, Translated Pertinent Survey Questions.
