# Supplementary material for: Managing Obesity in Lockdown: Survey of Health Behaviors and Telemedicine
Source: Nutrients. 2021 Apr 19;13(4):1359. doi: 10.3390/nu13041359 (PMC8073707; doi:10.3390/nu13041359)
Supplement: Supplementary file 1 [file nutrients-13-01359-s001.zip › Supplementary Materials.pdf]

Translated Pertinent Survey Questions

Informed consent:

1. I consent voluntarily to be a participant in this study, and I am aware of my right to stop my participation at any time without need for an explanation
    a. Yes
    b. No

Personal details

2. Age :
3. Gender:
    a. Male
    b. Female
    c. I would rather not say
4. Name of city or town in which you reside:
5. Please indicate your weight in kg as measured before the COVID-19 outbreak (skip if unknown):
6. Please indicate your current weight in kg:
7. Employment during lockdown:
    a. I did not work before and I do not work at the present
    b. I worked before and I am not working at the present
    c. I am working from home
    d. I am an essential worker working outside the home
    e. I am a student/soldier
    f. I am retired
8. Please mark the statements that apply to you:
    a. I received medications for weight management during the COVID-19 outbreak
    b. I underwent endoscopic sleeve gastroplasty
    c. I underwent bariatric surgery in the past year
    d. I underwent bariatric surgery over a year ago
    e. None of the above

Diet and physical activity

1. I feel during this period I have been eating:
    a. More than usual
    b. Less than usual
    c. No change
2. During this period, I have been eating processed snacks (sweet/savory):
    a. More than usual
    b. Less than usual
    c. No change
3. During this period, I have been eating fruits and vegetables:
    a. More than usual
    b. Less than usual
    c. No change
4. During the COVID-19 outbreak (mark all statements that apply to you):
    a. I ate more homemade food
    b. I ate more fast food
    c. I ate less homemade food
    d. I ate less fast food
    e. There was no change in my homemade food consumption
    f. There was no change in my fast food consumption
5. During this period:
    a. I adhere more to having organized and planned meals throughout the day
    b. I adhere less to having organized and planned meals throughout the day
    c. There is no change in my adherence to organized and planned meals throughout the day
6. Which of the following statements best describes your eating habits during the COVID-19 outbreak (mark all statements that apply to you):
    a. Eating more meals at the table
    b. Eating less meals at the table
    c. I live by myself
    d. Eating more often with household members

    e. Eating less often with household members

7. Before the COVID-19 outbreak: How much time (in hours) per week did you dedicate to physical activity?

    a. < 1

    b. 2

    c. 3

    d. 4

    e. 5

    f. > 5

8. During the COVID-19 outbreak: How much time (in hours per week) did you dedicate to physical activity?

    a. < 1

    b. 2

    c. 3

    d. 4

    e. > 5

Sleep and Mood

1. How did the COVID-19 crisis affect your sleeping hours?

    a. I sleep more hours on average every night as compared to my previous routine

    b. I sleep less hours on average every night as compare to my previous routine

    c. There is no change in number of hours I sleep at night

2. Was there nighttime eating (at late hours or at a time that you are supposed to be asleep) before the current period?

    a. Yes

    b. No

    c. Sometimes

    d. I do not know

3. Is there nighttime eating (at late hours or at a time that you are supposed to be asleep) currently?

    a. Yes

    b. No

    c. Sometimes

    d. I do not know

4. How has this period affected your mood?
    a. My mood improved
    b. My mood worsened
    c. There is no change in my mood
    d. I do not know

Virtual Care

1. Do you utilize the virtual care services available in our clinic?
    a. Yes, since the COVID-19 outbreak I have been receiving virtual consultations
    b. Yes, and I received virtual care prior to the COVID-19 outbreak as well
    c. I received virtual care prior to COVID-19 and I am not using it at the present
    d. No
2. I utilized virtual care for the following types of consultations (mark all applicable selections):
    a. Medical
    b. Dietary
    c. Psychological
3. Do you believe that you will continue to utilize the dietary online care after the COVID-19 crisis ends?
    a. Yes, certainly
    b. Yes, in part
    c. Not at all
    d. I do not know
